# Facile construction of Pt/TiO_2_/Se/Ni heterostructure for efficient visible-light-driven PEC water splitting

**DOI:** 10.3389/fchem.2025.1688359

**Published:** 2025-09-29

**Authors:** Ying-Chu Chen, Yen-Wei Huang, Yu-Kuei Hsu

**Affiliations:** 1 Department of Chemical Engineering & Biotechnology, National Taipei University of Technology, Taipei, Taiwan; 2 Department of Opto-Electronic Engineering, National Dong Hwa University, Hualien, Taiwan

**Keywords:** selenium, titanium oxide, platinum, photoelectrochemical, hydrogen

## Abstract

In this study, a novel Pt/TiO_2_/Se/Ni heterostructure photocathode was successfully fabricated via a simple and cost-effective method involving galvanic replacement, thermal annealing, and sequential spin-coating processes. Amorphous selenium was first deposited on nickel foil and subsequently transformed into crystalline trigonal Se through thermal treatment. The TiO_2_ and Pt nanoparticles were then uniformly decorated onto the Se surface to form a hierarchical heterostructure. Structural, morphological, and compositional characterizations using XRD, SEM, Raman spectroscopy, and XPS confirmed the formation of trigonal selenium and the successful deposition of TiO_2_ and Pt. Optical and photoelectrochemical (PEC) analyses revealed that the crystalline Se exhibited an optimal band gap of 1.89 eV and efficient visible light absorption. The Pt/TiO_2_/Se photocathode delivered a significantly enhanced photocurrent density of −5 mA cm^-2^ at −0.3 V vs. Ag/AgCl, which is 1.6 times higher than that of the bare Se electrode. Mott–Schottky and EIS analyses demonstrated an increased carrier density and reduced charge transfer resistance, facilitating efficient charge separation and transfer. These findings highlight the great potential of the Pt/TiO_2_/Se heterostructure as a high-performance photocathode for solar hydrogen production applications.

## 1 Introduction

The rapid economic development of many countries in recent decades has resulted in a significant increase in global energy consumption. However, the heavy reliance on fossil fuels for energy production has led to serious environmental issues, particularly in the form of pollution and greenhouse gas emissions. In response, the development and adoption of clean and sustainable energy sources have garnered widespread global attention. Among these, solar energy has emerged as a promising alternative to mitigate the adverse impacts of fossil fuel usage (Bak et al., 2002; Chen et al., 2021; Khaselev and Turner, 1998; Xiong et al., 2024). Photoelectrochemical (PEC) hydrogen production, which utilizes solar energy to generate hydrogen fuel, is one of the most promising strategies for renewable energy conversion. Extensive research has focused on the development of various semiconductor materials—such as oxides, sulfides, selenides, and tellurides—by tailoring their structures and tuning their bandgaps to enhance PEC performance (Chen et al., 2018a; Chen et al., 2018b; Hisatomi et al., 2014; Yang et al., 2024). Among these, chalcogenide semiconductors such as Se, CdS, and Sb_2_Se_3_ are promising candidates because of their narrow band gaps and strong visible-light absorption, which enable efficient utilization of the solar spectrum. However, their practical application is often hindered by poor stability and rapid surface oxidation under PEC operation. Selenium (Se), a group VI chalcogen element, has attracted considerable attention due to its unique allotropes and favorable optoelectronic properties. It exists in three stable allotropes: amorphous red Se (composed of Se_8_ rings), black Se (with polymeric Se_8_ chains), and trigonal gray Se (featuring helical chains arranged in a hexagonal lattice). Trigonal Se, in particular, exhibits a bandgap in the range of 1.6–2.0 eV and a lower electron affinity compared to sulfur or oxygen, making it a promising candidate for promoting the hydrogen evolution reaction (HER) (Jo et al., 2021). Despite these advantages, reports on the application of trigonal Se as a photoelectrode material for PEC hydrogen generation remain limited. For instance, Li et al. demonstrated a Se thin-film photocathode fabricated via thermal evaporation on an FTO substrate, which achieved a photocurrent density of −7.2 mA cm^-2^ at 0 V vs. RHE (Li et al., 2021). However, the complexity of the deposition processes, high equipment costs, and significant energy requirements pose challenges for practical application. In addition, TiO_2_ has been widely employed as a protective layer or interfacial mediator. Owing to its chemical stability, appropriate band alignment, and facile electron transport properties, TiO_2_ can effectively suppress photocorrosion of chalcogenides and facilitate charge separation at the heterojunction interface. Moreover, the incorporation of Pt nanoparticles as a cocatalyst has been demonstrated to significantly reduce the overpotential for HER and enhance charge transfer kinetics, owing to the superior catalytic activity of Pt toward hydrogen evolution. In this study, we present a facile and cost-effective strategy for fabricating Pt/TiO_2_/Se/Ni foil heterostructure photoelectrodes. Amorphous Se films were initially deposited on nickel foil via a galvanic displacement reaction, followed by vacuum thermal annealing to form crystalline trigonal Se. Subsequently, TiO_2_ and Pt nanoparticles (NPs) were deposited via spin-coating using colloidal solutions. The PEC performance of the resulting heterostructured photoelectrodes was systematically investigated. Notably, this straightforward fabrication method yielded a substantial photocurrent response, attributed to improved charge separation and accelerated interfacial charge transfer.

## 2 Experimental process

**FIGURE 1:**
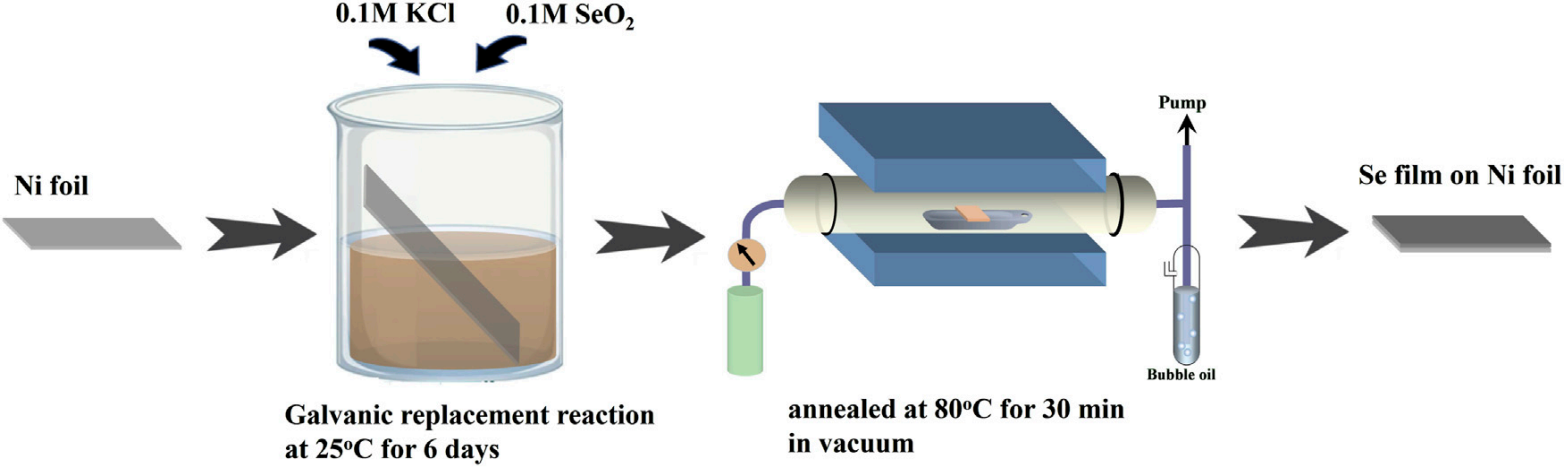
Schematic diagram of trigonal Se thin film synthesis.

All chemicals and reagents used in this study were of analytical grade and used without further purification. The synthesis process of crystalline selenium is illustrated in Figure 1. Nickel foil (1 × 2.5 cm^2^) was sequentially cleaned with acetone, deionized water, 0.5 M nitric acid, and again with deionized water, each step lasting for 15 min. The thoroughly cleaned foil was then immersed in an aqueous solution containing 0.1 M KCl and 0.1 M SeO_2_ for 6 days, allowing for the deposition of red amorphous selenium on the surface. Following deposition, the sample was placed at the center of a tubular furnace and thermally annealed at 80 °C for 30 min. After natural cooling to room temperature, the color of the sample turned gray, indicating the formation of trigonal selenium (Se/Ni foil). A TiO_2_ colloidal solution was prepared by mixing 0.1 M titanium isopropoxide with ethanol at 25 °C. This solution was spin-coated onto the surface of the Se/Ni foil. The same process was used to prepare TiO2/FTO for Raman spectroscopy measurements. For the deposition of Pt nanoparticles (NPs), a colloidal Pt solution was synthesized via chemical reduction. Briefly, a 1 mM aqueous solution of K_2_PtCl_6_ (30 mL) was heated to 100 °C, followed by the rapid addition of 2 mL of anhydrous methanol under vigorous stirring. The reaction was maintained at the boiling point for 2 h. The resulting Pt colloid was then spin-coated onto the TiO_2_/Se/Ni sample to fabricate the final Pt/TiO_2_/Se/Ni heterostructure. To complete the electrode preparation, a copper wire was attached to the edge of the sample using silver paste and cured at 80 °C for 20 min. The bonding area was subsequently sealed with epoxy resin, leaving only the active surface exposed for electrochemical testing.

The surface morphology of the Pt/TiO_2_/Se/Ni heterostructure was characterized using scanning electron microscopy (SEM, JEM-4000EX). Crystalline structure was examined via X-ray diffraction (XRD, Bruker D8 Advance) employing Cu Kα radiation (λ = 0.1506 nm). The elemental composition and chemical states were analyzed by X-ray photoelectron spectroscopy (XPS, Perkin-Elmer PHI 1600). Photoelectrochemical (PEC) measurements were carried out using a CHI 627D potentiostat in a 0.1 M H_2_SO_4_ electrolyte (pH ≈ 1). A conventional three-electrode setup was employed, consisting of the Pt/TiO_2_/Se/Ni foil as the working electrode, a platinum foil as the counter electrode, and an Ag/AgCl (in 3 M KCl) reference electrode. Illumination was provided by a 150 W xenon (Xe) lamp with an AM 1.5 filter, delivering a light intensity of 100 mW cm^-2^ at the sample surface. For incident photon-to-electron conversion efficiency (IPCE) measurements, a 150 W Xe lamp coupled with a monochromator was used to provide monochromatic light. Mott–Schottky (M–S) analysis was conducted in the dark over a potential range from 0.05 to −0.3 V vs. Ag/AgCl at a frequency of 10 kHz with a 10 mV AC amplitude.

## 3 Results and discussion

### 3.1 Material analysis

**FIGURE 2:**
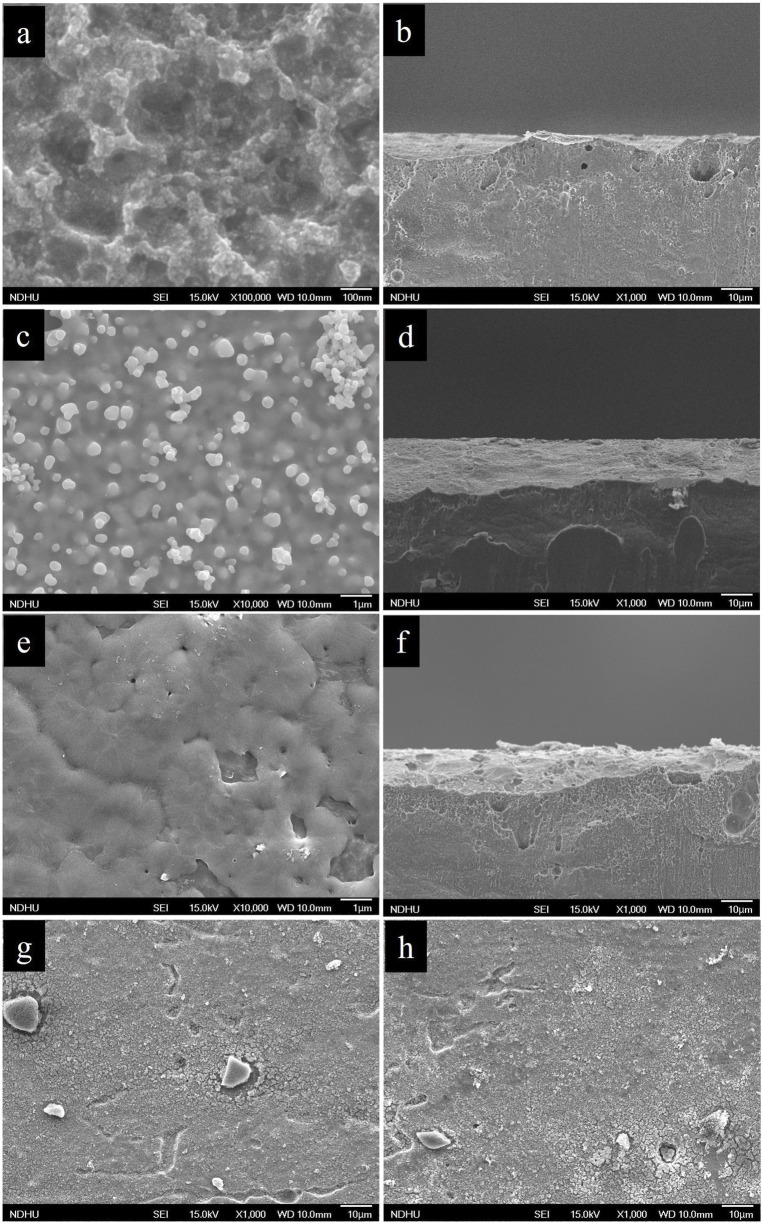
FESEM of **(a) (b)** bare Ni foil, **(c) (d)** as-grown Se, **(e) (f)** annealed Se, **(g)** TiO_2_/Se, **(h)** Pt/TiO_2_/Se samples.

The galvanic replacement reaction was conducted by immersing pre-cleaned nickel substrates into an aqueous solution of 0.1 M SeO_2_. In neutral or mildly alkaline environments, SeO_2_ readily dissolves and ionizes to form SeO_3_^2-^ and HSeO_3_^−^ species (Xu et al., 2015). Notably, the standard reduction potential of the SeO_3_^2-^/Se redox couple (0.74 V vs. standard hydrogen electrode, SHE) is significantly higher than that of the Ni^2+^/Ni couple (−0.25 V vs. SHE) (Tendenedzai et al., 2021), making the redox process thermodynamically favorable. The spontaneous redox reaction proceeds according to the following Equation 1:

$${\text{SeO}}_{3}^{2-}+2\text{Ni}+6H^{+}\;\to \text{ Se}+3{\text{Ni}}^{2+}+3H_{2}O$$ (1)

where Ni atoms are oxidized and dissolve into the solution as Ni^2+^, while Se is reduced and deposited onto the Ni substrate in the form of a nanostructured film. Figure 2 illustrates the morphological evolution of the Pt/TiO_2_/Se/Ni foil at various stages of fabrication. Initially, the bare Ni foil exhibits a rough and irregular surface morphology due to the removal of its native oxide layer (Figures 2a,b). Upon completion of the galvanic displacement reaction, a uniform amorphous Se layer composed of aggregated granular nanostructures is formed (Figures 2c,d). This Se film has a thickness of approximately 200 nm and uniformly covers the Ni substrate. The Se layer prepared by galvanic replacement reaction is not fully dense but partially porous, which allows electrolyte penetration and ensures the galvanic replacement reaction can be sustained on the underlying Ni surface. This porous nature facilitates continuous Se growth while preventing complete passivation of the Ni substrate. After thermal annealing, the granular features are replaced by a dense, continuous crystalline Se film, indicating a successful phase transition to trigonal selenium (Figures 2e,f). Subsequent spin-coating of TiO_2_ results in a roughened surface texture, confirming the formation of a Se/TiO_2_ hierarchical nanostructure (Figure 2g). Finally, the deposition of Pt nanoparticles has little impact on the overall morphology due to their low loading and small particle size, allowing the underlying nanostructure to remain largely intact (Figure 2h).

**FIGURE 3:**
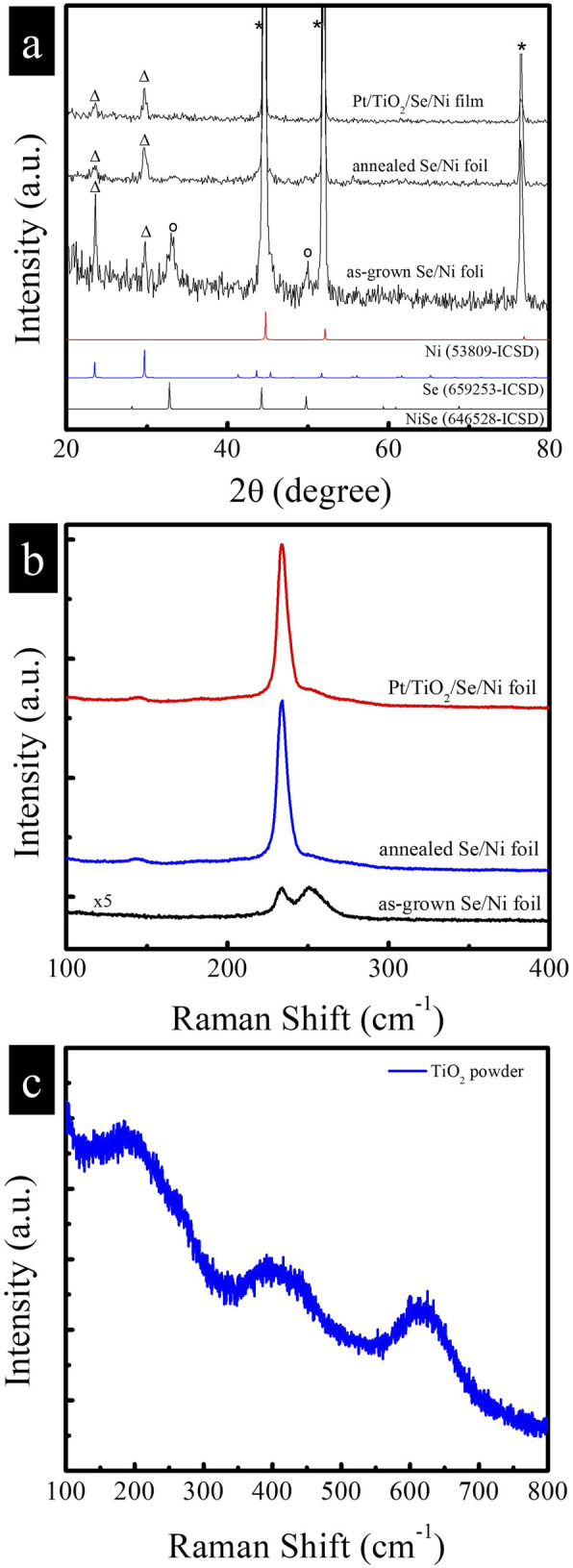
**(a)** X-ray diffraction patterns and **(b)** Raman spectra of as-grown Se, annealed Se, and Pt/TiO2/Se samples. **(c)** Raman spectra of TiO2 powder sample.

The crystalline phases of the as-grown selenium, as well as the samples after thermal treatment and subsequent decoration with TiO_2_ and Pt nanoparticles (NPs), were characterized by powder X-ray diffraction (XRD), as shown in Figure 3a. The XRD pattern of the as-deposited sample obtained via galvanic replacement reveals the presence of weak diffraction peaks corresponding to trigonal selenium (ICSD No. 659253, marked with triangles), along with prominent peaks from the underlying Ni foil substrate (marked with inverted stars). In addition, diffraction peaks associated with nickel selenide (NiSe, ICSD No. 646528, marked with circles) are observed, which likely formed due to the reaction between the Se precursor and Ni^2+^ ions released during the galvanic process. To further confirm the phase composition, Raman spectroscopy was employed, and the results are shown in Figure 3b. The as-grown film displays a broad band centered around ∼250 cm^-1^, characteristic of disordered selenium chains with a minor contribution from Se_8_ rings. A shoulder observed at ∼235 cm^-1^ indicates the partial presence of trigonal selenium crystallites embedded within the amorphous matrix (Nagata et al., 1981). Following thermal annealing, the XRD pattern exhibits significantly intensified peaks corresponding to the trigonal phase of selenium, while the NiSe-related peaks become negligible. This transformation is corroborated by the Raman spectrum, which shows a pronounced vibrational mode at 235 cm^-1^, characteristic of crystalline trigonal selenium, thereby confirming successful phase conversion. Upon thermal annealing in Se vapor, NiSe is unstable and undergoes phase transformation due to the outward diffusion of Ni and concurrent recrystallization of Se. This process leads to the disappearance of NiSe peaks in XRD/Raman spectra, leaving predominantly trigonal Se. After deposition of TiO_2_ and Pt nanoparticles via spin-coating, no distinct diffraction peaks corresponding to TiO_2_ or Pt are observed in the XRD patterns. This absence is attributed to their relatively low loading and the nanoscale dimensions of the deposited materials. However, to confirm the crystallographic phase of the TiO_2_, a reference sample was prepared by depositing the same TiO_2_ colloidal solution onto a fluorine-doped tin oxide (FTO) substrate. As shown in Figure 3c, the Raman spectrum of the TiO_2_/FTO sample reveals diffraction peaks that correspond to the anatase phase of TiO_2_, confirming the identity of the deposited material.

**FIGURE 4:**
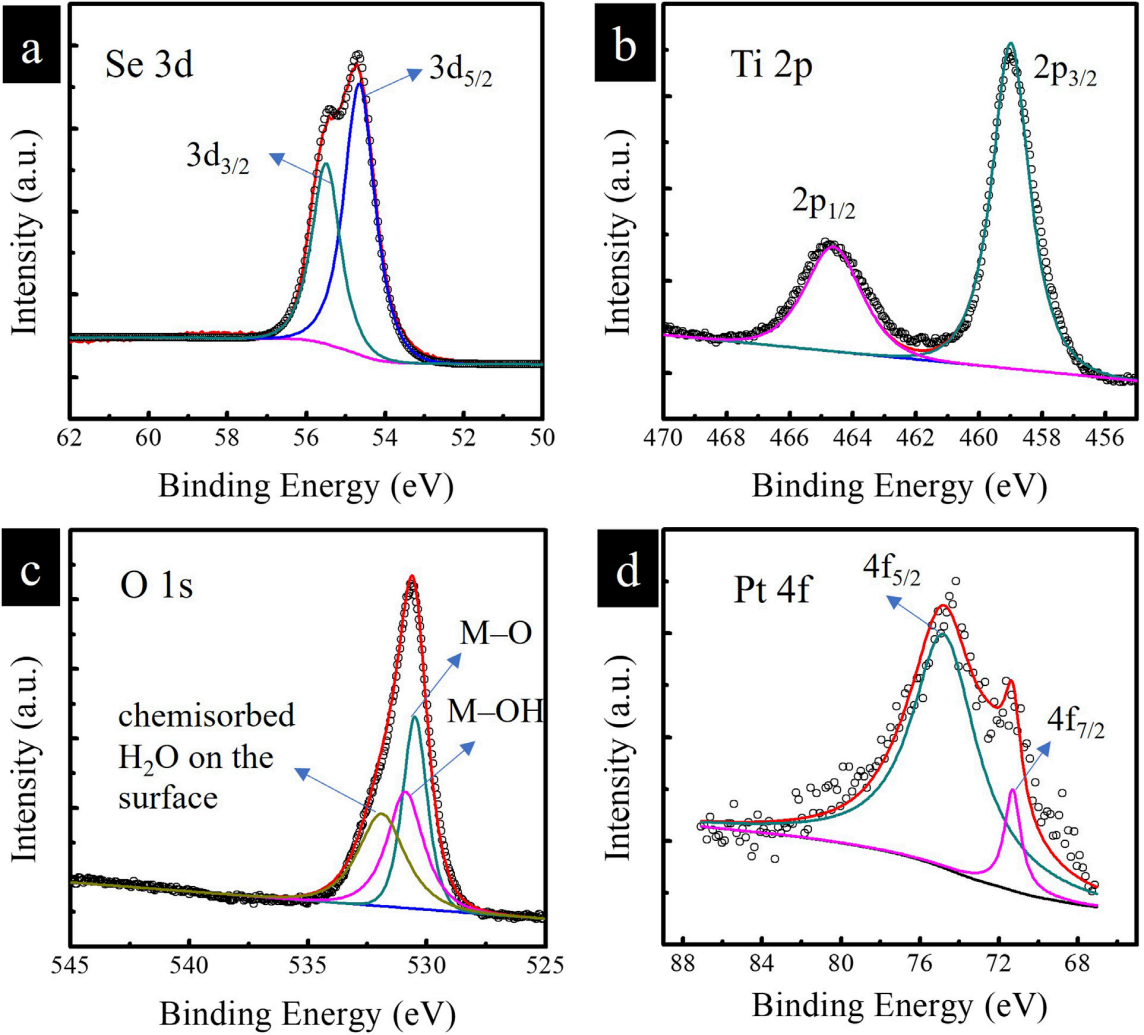
**(a)** Se 3d, **(b)** Ti 2p, **(c)** O1s, and **(d)** Pt 4f of the Pt/TiO_2_/Se sample.

**FIGURE 5:**
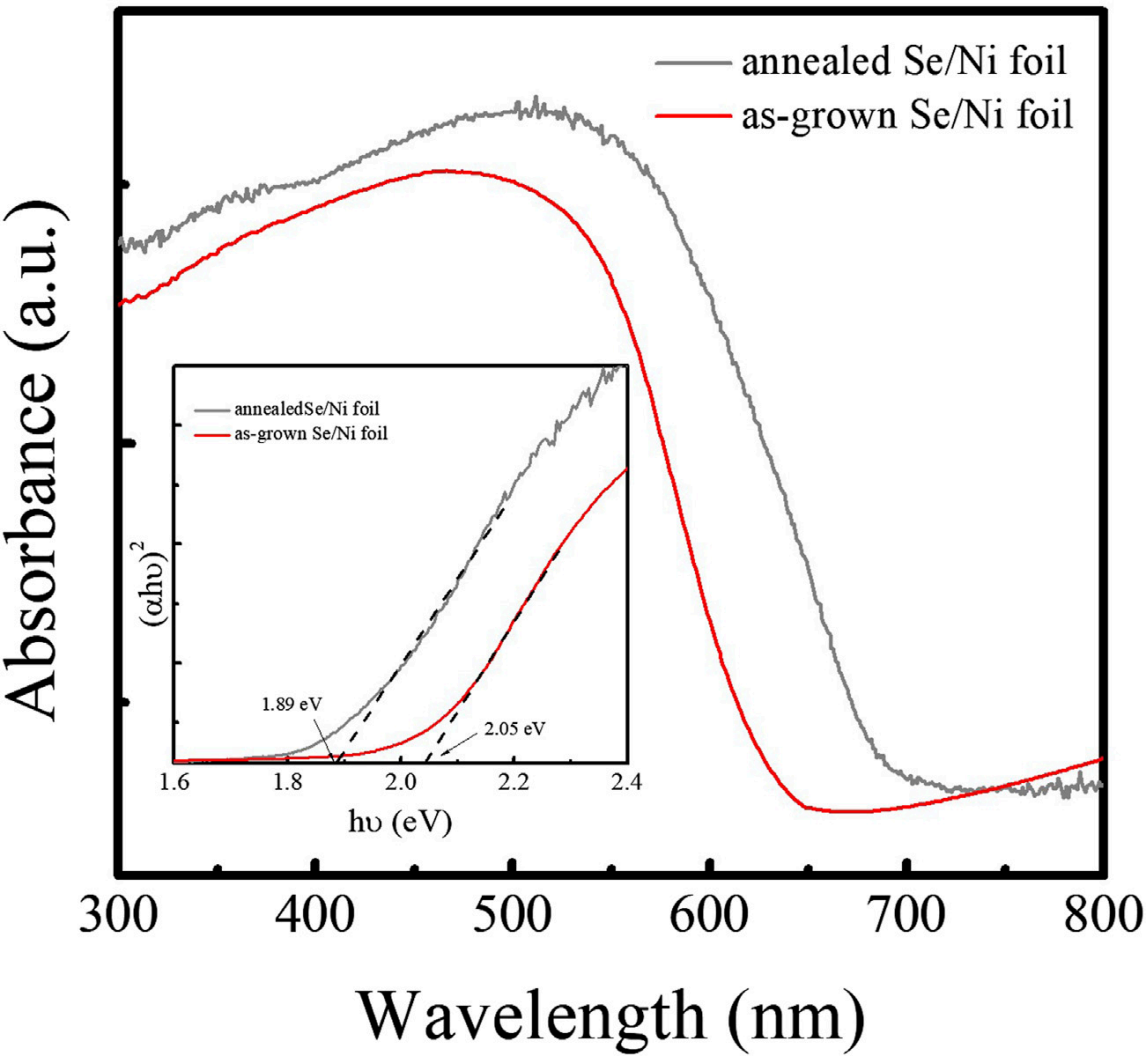
Absorption spectra of as-grown and annealed Se samples.

X-ray photoelectron spectroscopy (XPS) was employed to analyze the chemical states of the elements present in the Pt/TiO_2_/Se heterostructure. The high-resolution spectra of Se 3d, Ti 2p, O 1s, and Pt 4f are shown in Figure 4. The Se 3d spectrum displays two peaks at binding energies of 54.68 eV (3d_5_/_2_) and 55.53 eV (3d_3_/_2_), separated by 0.85 eV with an area ratio close to 1.5 and full width at half maximum (FWHM) values around 0.65 eV. These features are characteristic of elemental selenium (Se^0^) (Figure 4a) (Ruiz-Fresneda et al., 2020). The Ti 2p spectrum exhibits two peaks located at 458.9 eV and 464.6 eV, corresponding to the Ti 2p_3_/_2_ and Ti 2p_1_/_2_ spin–orbit components, respectively (Figure 4b). These peaks are indicative of the Ti^4+^ oxidation state in TiO_2_ (Yang et al., 2024). The O 1s spectrum can be deconvoluted into three components centered at 530.5 eV, 530.9 eV, and 531.8 eV (Figure 4c). The peaks at 530.5 and 530.9 eV are attributed to metal–oxygen (M–O) and metal–hydroxyl (M–OH) bonds, respectively (Chen et al., 2020), while the peak at 531.8 eV is associated with chemisorbed water molecules on the surface. The Pt 4f core level region reveals two peaks at 71.3 eV and 74.8 eV, corresponding to the Pt 4f_7_/_2_ and Pt 4f_5_/_2_ levels of metallic platinum (Pt^0^), confirming the successful deposition of elemental Pt (Figure 4d; Yang et al., 2024). These XPS results are in good agreement with the phase information obtained from XRD and Raman spectroscopy, further validating the chemical composition and structure of the Pt/TiO_2_/Se heterostructure. In addition, optical absorption properties of the samples were investigated using UV–vis spectroscopy. The Tauc plots, constructed by plotting (αhν)^2^ versus photon energy (hν) in the absorption edge region, are shown in Figure 5. The optical bandgap of the as-deposited amorphous Se was estimated to be 2.05 eV, whereas that of the thermally treated sample decreased to 1.89 eV. The reduction in bandgap is attributed to the phase transformation from amorphous to trigonal selenium. These bandgap values are consistent with those previously reported for selenium-based materials (Li et al., 2021).

### 3.2 Photoelectrochemical performance

**FIGURE 6:**
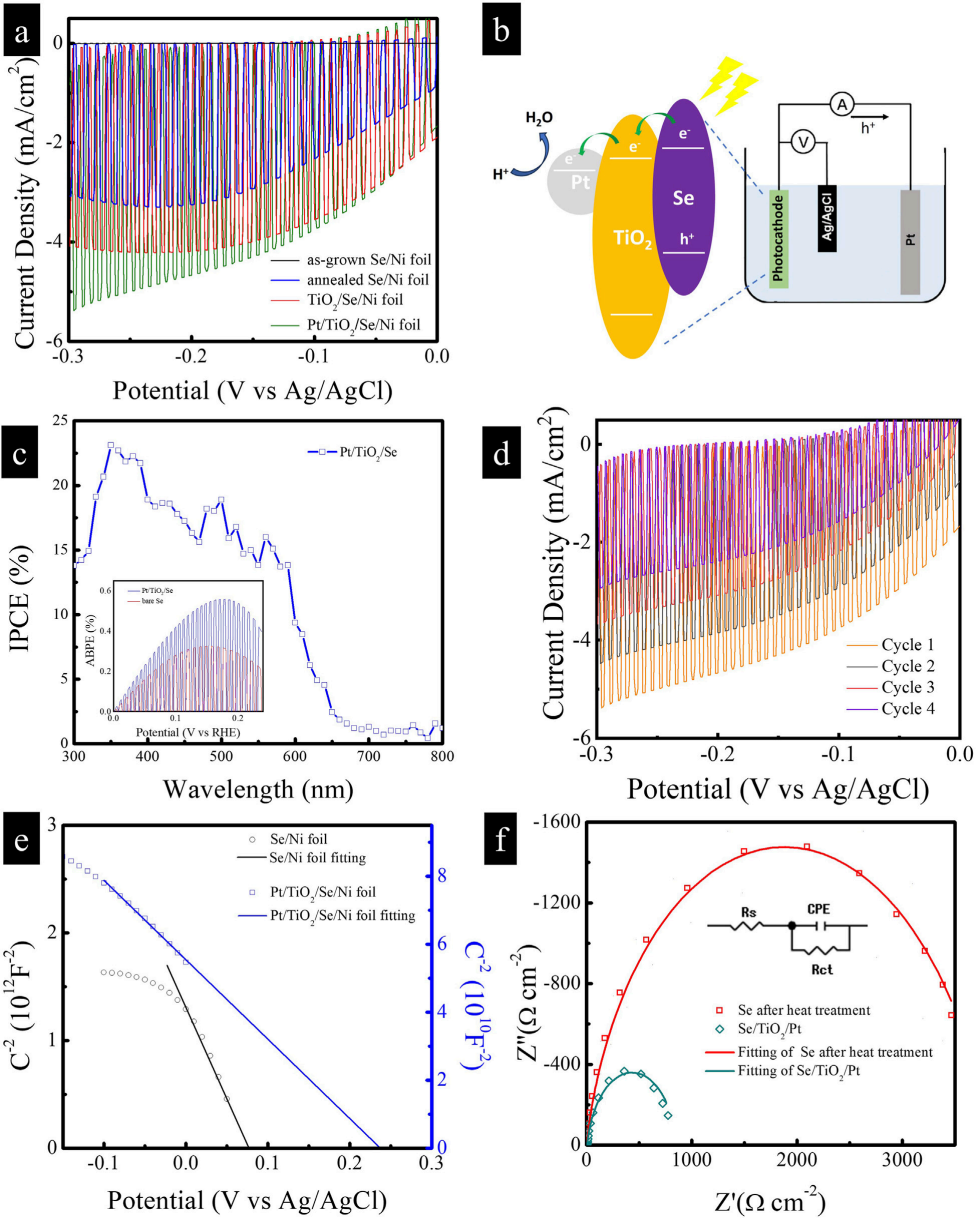
**(a)** LSV of as-grown Se, annealed Se, TiO_2_/Se, and Pt/TiO_2_/Se samples. **(b)** Scheme diagram of charge transfer pathway in the Pt/TiO_2_/Se heterostructure. **(c)** IPCE of Pt/TiO_2_/Se sample, inset is ABPE. **(d)** The stability of the Pt/TiO_2_/Se photocathode by performing successive LSV cycles under illumination. **(e)** Mott-Schottky plots of annealed Se and Pt/TiO_2_/Se samples in dark, **(f)** EIS of annealed Se and Pt/TiO_2_/Se samples under illumination.

To evaluate the photoelectrochemical (PEC) performance of the Pt/TiO_2_/Se heterostructure electrode as a photocathode for solar-driven hydrogen generation via water splitting, measurements were conducted using a conventional three-electrode setup. The linear sweep voltammetry (LSV) curves are shown in Figure 6a. The as-deposited amorphous Se sample exhibited only a weak photocurrent. However, after thermal treatment, the trigonal Se electrode demonstrated a significantly enhanced cathodic photocurrent of −3 mA cm^-2^ at −0.3 V vs. Ag/AgCl. This photocurrent arises from the photo-induced reduction of water, in which photogenerated electrons participate in the hydrogen evolution reaction (HER). Following the sequential deposition of TiO_2_ and Pt nanoparticles, the photocurrent of the Pt/TiO_2_/Se electrode further increased to −5 mA cm^-2^ under the same potential, representing a ∼1.6-fold enhancement compared to the trigonal Se sample. As Pt is a noble metal with negligible light absorption, it cannot generate a measurable photocurrent under illumination. Similarly, in our study the amount of deposited TiO_2_ and Pt was very small, as confirmed by the SEM results (Figure 2). Therefore, the photocathodic current contributions from Pt and TiO_2_ alone are negligible. The observed photocurrent enhancement in the Pt/TiO_2_/Se/Ni heterostructure arises primarily from Se as the light-absorbing semiconductor, while TiO_2_ facilitates charge separation and Pt provides efficient catalytic sites for the hydrogen evolution reaction. Upon illumination, selenium (Se) absorbs visible light and generates electron–hole pairs, with photoexcited electrons promoted from the valence band (VB) to the conduction band (CB), as shown in Figure 6b. The photogenerated electrons are subsequently transferred to the CB of TiO_2_ owing to the favorable band alignment, which effectively suppresses electron–hole recombination in Se. These electrons are then efficiently delivered to Pt nanoparticles, which serve as active catalytic sites for the hydrogen evolution reaction (HER, 2H^+^ + 2e^−^ → H_2_). Meanwhile, the remaining holes in the VB of Se are consumed in oxidation reactions, thereby maintaining charge neutrality. This stepwise electron transfer pathway across the Se/TiO_2_/Pt interfaces facilitates spatial charge separation, accelerates interfacial charge migration, and significantly enhances the overall photoelectrochemical hydrogen generation efficiency. This improvement is attributed to both the enhanced crystallinity of Se after annealing and the synergistic effects of TiO_2_ and Pt NPs, which facilitate charge separation and reduce the overpotential for HER. Figure 6c presents the incident photon-to-electron conversion efficiency (IPCE) spectrum of the Pt/TiO_2_/Se photocathode at −0.3 V vs. Ag/AgCl. The device shows a photoresponse extending to ∼680 nm, consistent with the optical absorption edge of Se, confirming its ability to harvest visible light and contribute to PEC activity in that spectral region. The applied bias photon-to-current efficiency (ABPE) of the photocathodes was further calculated, and the ABPE values are presented in the inset of Figure 6c. The results confirm that the Pt/TiO_2_/Se/Ni heterostructure exhibits significantly higher ABPE compared with bare Se, demonstrating its superior efficiency for photoelectrochemical activity. However, the ABPE efficiency of the current Pt/TiO_2_/Se/Ni heterostructure is low, and thus the electrode in its present study is not directly applicable to practical hydrogen production. The focus of this work is to demonstrate the feasibility of constructing Se-based heterostructures via a facile galvanic replacement method and to elucidate the charge transfer mechanism at the Pt/TiO_2_/Se interface. In practical applications, combining the photovoltage of a photovoltaic device with the photocurrent of a Se photocathode in a tandem or solar cell-driven PEC structure could significantly improve the overall efficiency of hydrogen production systems. Importantly, compared to other Se-based photocathodes, the photocurrent density achieved in this study (−5 mA cm^-2^ at −0.3 V vs. Ag/AgCl) is moderate. For instance, Li et al. reported a TiO_2_/Se/Pt photocathode prepared by thermal evaporation of Se followed by ALD of TiO_2_ and sputtering of Pt nanoparticles, which delivered a maximum photocurrent of ∼15 mA cm^-2^ (Li et al., 2021). This indicates that further optimization of the Se deposition and interfacial engineering could substantially improve the PEC performance of our system. The stability of the Pt/TiO_2_/Se photocathode was evaluated by performing successive LSV cycles under illumination. As shown in Figure 6d, the photocurrent was observed to gradually decrease with increasing cycle number, indicating limited long-term stability. This instability is mainly attributed to the insufficient compactness and completeness of the TiO_2_ protective layer prepared by the simple chemical method, which left portions of the Se electrode surface exposed to the electrolyte. To overcome this issue, we suggest that atomic layer deposition (ALD) of TiO_2_ could be employed to form a dense and conformal protective coating. Such an approach is expected to significantly enhance the durability of the photocathode under prolonged photoelectrochemical operation. Incorporation of other protective overlayers such as Al_2_O_3_ or NiOx, as well as catalyst engineering, may also further enhance the durability of the photocathode. Nevertheless, the stability observed in our study is comparable to that reported for similar Se-based systems. For example, Li et al. demonstrated that their TiO_2_/Se/Pt photocathode exhibited an initial photocurrent of ∼6.5 mA cm^-2^, which decayed to ∼1.5 mA cm^-2^ after 7,000 s of continuous operation (Li et al., 2021). This highlights that despite different preparation routes, Se photocathodes generally suffer from limited stability under prolonged illumination, and further advances in protective coating design and catalyst integration will be required to achieve long-term operation. Mott–Schottky (M–S) analysis was performed in the dark to investigate the charge carrier properties of the samples, including the as-grown Se and the modified electrodes after thermal treatment and TiO_2_ deposition. To minimize the formation of surface oxide layers, the applied potential window was carefully restricted to −0.15 V to +0.05 V versus Ag/AgCl. Within this range, the electrochemical stability of selenium is preserved, and the risk of oxide formation is negligible. Capacitance values were extracted from electrochemical impedance spectroscopy (EIS) data obtained at 10 kHz with an AC amplitude of 10 mV. As shown in Figure 6e, the M–S plots exhibit a negative slope, confirming the p-type semiconductor behavior of Se. According to the Mott–Schottky Equation 2:

$$\frac{1}{C^{2}}=\left(\frac{2}{e\varepsilon \varepsilon_{0}N_{a}}\right)\left[V-V_{FB}-\frac{k_{B}T}{e}\right]$$ (2)

where C is the space charge capacitance, e is the elementary charge, ε is the dielectric constant of Se (ε = 6), ε_0_ is the vacuum permittivity, N_a_ is the acceptor concentration, V is the applied potential, V_FB_ is the flat-band potential, T is the temperature, and k_B_ is the Boltzmann constant. From the linear fitting of the plots, the flat-band potentials were estimated to be 0.08 V and 0.24 V vs. Ag/AgCl for the bare Se and Pt/TiO_2_/Se electrodes, respectively. The corresponding carrier concentrations were calculated to be 4.7 × 10^17^ cm^-3^ for the bare Se and 9.6 × 10^19^ cm^-3^ for the Pt/TiO_2_/Se sample. The significantly higher carrier density in the modified electrode leads to a stronger internal electric field in the depletion region, thereby promoting more efficient separation and transport of photogenerated charge carriers. Furthermore, electrochemical impedance spectroscopy (EIS) was employed to evaluate the interfacial charge transfer characteristics. As shown in Figure 6f, the Nyquist plot was fitted using a complex nonlinear least squares (CNLS) method based on an equivalent circuit (inset). The charge transfer resistance (R_ct_) of the Pt/TiO_2_/Se electrode was determined to be 851 Ω cm^-2^, indicating efficient charge injection from the heterojunction to the electrolyte. These results collectively demonstrate that the Pt/TiO_2_/Se heterostructure exhibits superior PEC performance compared to bare Se, owing to enhanced light absorption, improved charge carrier mobility, and reduced interfacial resistance. The uniform decoration of TiO_2_ and Pt NPs provides an effective strategy for engineering high-performance photocathodes for solar hydrogen production.

## 4 Conclusion

In summary, a hierarchical Pt/TiO_2_/Se/Ni heterostructure electrode was successfully developed through a straightforward and scalable fabrication process. The conversion of amorphous selenium to its trigonal crystalline phase via thermal annealing significantly improved its optoelectronic properties. The subsequent decoration with TiO_2_ and Pt nanoparticles enhanced both charge separation and surface catalytic activity. The optimized Pt/TiO_2_/Se photocathode exhibited a photocurrent density of −5 mA cm^-2^ at −0.3 V vs. Ag/AgCl, along with a high IPCE response extending to 680 nm. Mott–Schottky analysis confirmed a substantial increase in carrier concentration and a favorable flat-band potential, while EIS measurements revealed reduced charge transfer resistance. These enhancements synergistically contribute to the superior PEC performance of the heterostructure. The results demonstrate that the rational design of Se-based heterostructures with semiconductor and metal co-catalyst components represents a promising strategy for efficient solar-driven hydrogen generation.

## Data Availability

The original contributions presented in the study are included in the article/supplementary material, further inquiries can be directed to the corresponding author.
